# Evaluating the Effects of SARS-CoV-2 Spike Mutation D614G on Transmissibility and Pathogenicity

**DOI:** 10.1016/j.cell.2020.11.020

**Published:** 2021-01-07

**Authors:** Erik Volz, Verity Hill, John T. McCrone, Anna Price, David Jorgensen, Áine O’Toole, Joel Southgate, Robert Johnson, Ben Jackson, Fabricia F. Nascimento, Sara M. Rey, Samuel M. Nicholls, Rachel M. Colquhoun, Ana da Silva Filipe, James Shepherd, David J. Pascall, Rajiv Shah, Natasha Jesudason, Kathy Li, Ruth Jarrett, Nicole Pacchiarini, Matthew Bull, Lily Geidelberg, Igor Siveroni, COG-UK Consortium, Cherian Koshy, Emma Wise, Nick Cortes, Jessica Lynch, Stephen Kidd, Matilde Mori, Derek J. Fairley, Tanya Curran, James P. McKenna, Helen Adams, Christophe Fraser, Tanya Golubchik, David Bonsall, Catrin Moore, Sarah L. Caddy, Fahad A. Khokhar, Michelle Wantoch, Nicola Reynolds, Ben Warne, Joshua Maksimovic, Karla Spellman, Kathryn McCluggage, Michaela John, Robert Beer, Safiah Afifi, Sian Morgan, Angela Marchbank, Anna Price, Christine Kitchen, Huw Gulliver, Ian Merrick, Joel Southgate, Martyn Guest, Robert Munn, Trudy Workman, Thomas R. Connor, William Fuller, Catherine Bresner, Luke B. Snell, Themoula Charalampous, Gaia Nebbia, Rahul Batra, Jonathan Edgeworth, Samuel C. Robson, Angela Beckett, Katie F. Loveson, David M. Aanensen, Anthony P. Underwood, Corin A. Yeats, Khalil Abudahab, Ben E.W. Taylor, Mirko Menegazzo, Gemma Clark, Wendy Smith, Manjinder Khakh, Vicki M. Fleming, Michelle M. Lister, Hannah C. Howson-Wells, Louise Berry, Tim Boswell, Amelia Joseph, Iona Willingham, Paul Bird, Thomas Helmer, Karlie Fallon, Christopher Holmes, Julian Tang, Veena Raviprakash, Sharon Campbell, Nicola Sheriff, Matthew W. Loose, Nadine Holmes, Christopher Moore, Matthew Carlile, Victoria Wright, Fei Sang, Johnny Debebe, Francesc Coll, Adrian W. Signell, Gilberto Betancor, Harry D. Wilson, Theresa Feltwell, Charlotte J. Houldcroft, Sahar Eldirdiri, Anita Kenyon, Thomas Davis, Oliver Pybus, Louis du Plessis, Alex Zarebski, Jayna Raghwani, Moritz Kraemer, Sarah Francois, Stephen Attwood, Tetyana Vasylyeva, M. Estee Torok, William L. Hamilton, Ian G. Goodfellow, Grant Hall, Aminu S. Jahun, Yasmin Chaudhry, Myra Hosmillo, Malte L. Pinckert, Iliana Georgana, Anna Yakovleva, Luke W. Meredith, Samuel Moses, Hannah Lowe, Felicity Ryan, Chloe L. Fisher, Ali R. Awan, John Boyes, Judith Breuer, Kathryn Ann Harris, Julianne Rose Brown, Divya Shah, Laura Atkinson, Jack C.D. Lee, Adela Alcolea-Medina, Nathan Moore, Nicholas Cortes, Rebecca Williams, Michael R. Chapman, Lisa J. Levett, Judith Heaney, Darren L. Smith, Matthew Bashton, Gregory R. Young, John Allan, Joshua Loh, Paul A. Randell, Alison Cox, Pinglawathee Madona, Alison Holmes, Frances Bolt, James Price, Siddharth Mookerjee, Aileen Rowan, Graham P. Taylor, Manon Ragonnet-Cronin, Fabricia F. Nascimento, David Jorgensen, Igor Siveroni, Rob Johnson, Olivia Boyd, Lily Geidelberg, Erik M. Volz, Kirstyn Brunker, Katherine L. Smollett, Nicholas J. Loman, Joshua Quick, Claire McMurray, Joanne Stockton, Sam Nicholls, Will Rowe, Radoslaw Poplawski, Rocio T. Martinez-Nunez, Jenifer Mason, Trevor I. Robinson, Elaine O'Toole, Joanne Watts, Cassie Breen, Angela Cowell, Catherine Ludden, Graciela Sluga, Nicholas W. Machin, Shazaad S.Y. Ahmad, Ryan P. George, Fenella Halstead, Venkat Sivaprakasam, Emma C. Thomson, James G. Shepherd, Patawee Asamaphan, Marc O. Niebel, Kathy K. Li, Rajiv N. Shah, Natasha G. Jesudason, Yasmin A. Parr, Lily Tong, Alice Broos, Daniel Mair, Jenna Nichols, Stephen N. Carmichael, Kyriaki Nomikou, Elihu Aranday-Cortes, Natasha Johnson, Igor Starinskij, Ana da Silva Filipe, David L. Robertson, Richard J. Orton, Joseph Hughes, Sreenu Vattipally, Joshua B. Singer, Antony D. Hale, Louissa R. Macfarlane-Smith, Katherine L. Harper, Yusri Taha, Brendan A.I. Payne, Shirelle Burton-Fanning, Sheila Waugh, Jennifer Collins, Gary Eltringham, Kate E. Templeton, Martin P. McHugh, Rebecca Dewar, Elizabeth Wastenge, Samir Dervisevic, Rachael Stanley, Reenesh Prakash, Claire Stuart, Ngozi Elumogo, Dheeraj K. Sethi, Emma J. Meader, Lindsay J. Coupland, Will Potter, Clive Graham, Edward Barton, Debra Padgett, Garren Scott, Emma Swindells, Jane Greenaway, Andrew Nelson, Wen C. Yew, Paola C. Resende Silva, Monique Andersson, Robert Shaw, Timothy Peto, Anita Justice, David Eyre, Derrick Crooke, Sarah Hoosdally, Tim J. Sloan, Nichola Duckworth, Sarah Walsh, Anoop J. Chauhan, Sharon Glaysher, Kelly Bicknell, Sarah Wyllie, Ethan Butcher, Scott Elliott, Allyson Lloyd, Robert Impey, Nick Levene, Lynn Monaghan, Declan T. Bradley, Elias Allara, Clare Pearson, Peter Muir, Ian B. Vipond, Richard Hopes, Hannah M. Pymont, Stephanie Hutchings, Martin D. Curran, Surendra Parmar, Angie Lackenby, Tamyo Mbisa, Steven Platt, Shahjahan Miah, David Bibby, Carmen Manso, Jonathan Hubb, Meera Chand, Gavin Dabrera, Mary Ramsay, Daniel Bradshaw, Alicia Thornton, Richard Myers, Ulf Schaefer, Natalie Groves, Eileen Gallagher, David Lee, David Williams, Nicholas Ellaby, Ian Harrison, Hassan Hartman, Nikos Manesis, Vineet Patel, Chloe Bishop, Vicki Chalker, Husam Osman, Andrew Bosworth, Esther Robinson, Matthew T.G. Holden, Sharif Shaaban, Alec Birchley, Alexander Adams, Alisha Davies, Amy Gaskin, Amy Plimmer, Bree Gatica-Wilcox, Caoimhe McKerr, Catherine Moore, Chris Williams, David Heyburn, Elen De Lacy, Ember Hilvers, Fatima Downing, Giri Shankar, Hannah Jones, Hibo Asad, Jason Coombes, Joanne Watkins, Johnathan M. Evans, Laia Fina, Laura Gifford, Lauren Gilbert, Lee Graham, Malorie Perry, Mari Morgan, Matthew Bull, Michelle Cronin, Nicole Pacchiarini, Noel Craine, Rachel Jones, Robin Howe, Sally Corden, Sara Rey, Sara Kumziene-Summerhayes, Sarah Taylor, Simon Cottrell, Sophie Jones, Sue Edwards, Justin O’Grady, Andrew J. Page, John Wain, Mark A. Webber, Alison E. Mather, David J. Baker, Steven Rudder, Muhammad Yasir, Nicholas M. Thomson, Alp Aydin, Ana P. Tedim, Gemma L. Kay, Alexander J. Trotter, Rachel A.J. Gilroy, Nabil-Fareed Alikhan, Leonardo de Oliveira Martins, Thanh Le-Viet, Lizzie Meadows, Anastasia Kolyva, Maria Diaz, Andrew Bell, Ana Victoria Gutierrez, Ian G. Charles, Evelien M. Adriaenssens, Robert A. Kingsley, Anna Casey, David A. Simpson, Zoltan Molnar, Thomas Thompson, Erwan Acheson, Jane A.H. Masoli, Bridget A. Knight, Andrew Hattersley, Sian Ellard, Cressida Auckland, Tabitha W. Mahungu, Dianne Irish-Tavares, Tanzina Haque, Yann Bourgeois, Garry P. Scarlett, David G. Partridge, Mohammad Raza, Cariad Evans, Kate Johnson, Steven Liggett, Paul Baker, Sarah Essex, Ronan A. Lyons, Laura G. Caller, Sergi Castellano, Rachel J. Williams, Mark Kristiansen, Sunando Roy, Charlotte A. Williams, Patricia L. Dyal, Helena J. Tutill, Yasmin N. Panchbhaya, Leysa M. Forrest, Paola Niola, Jacqueline Findlay, Tony T. Brooks, Artemis Gavriil, Lamia Mestek-Boukhibar, Sam Weeks, Sarojini Pandey, Lisa Berry, Katie Jones, Alex Richter, Andrew Beggs, Colin P. Smith, Giselda Bucca, Andrew R. Hesketh, Ewan M. Harrison, Sharon J. Peacock, Sophie Palmer, Carol M. Churcher, Katherine L. Bellis, Sophia T. Girgis, Plamena Naydenova, Beth Blane, Sushmita Sridhar, Chris Ruis, Sally Forrest, Claire Cormie, Harmeet K. Gill, Joana Dias, Ellen E. Higginson, Mailis Maes, Jamie Young, Leanne M. Kermack, Nazreen F. Hadjirin, Dinesh Aggarwal, Luke Griffith, Tracey Swingler, Rose K. Davidson, Andrew Rambaut, Thomas Williams, Carlos E. Balcazar, Michael D. Gallagher, Áine O'Toole, Stefan Rooke, Ben Jackson, Rachel Colquhoun, Jordan Ashworth, Verity Hill, J.T. McCrone, Emily Scher, Xiaoyu Yu, Kathleen A. Williamson, Thomas D. Stanton, Stephen L. Michell, Claire M. Bewshea, Ben Temperton, Michelle L. Michelsen, Joanna Warwick-Dugdale, Robin Manley, Audrey Farbos, James W. Harrison, Christine M. Sambles, David J. Studholme, Aaron R. Jeffries, Alistair C. Darby, Julian A. Hiscox, Steve Paterson, Miren Iturriza-Gomara, Kathryn A. Jackson, Anita O. Lucaci, Edith E. Vamos, Margaret Hughes, Lucille Rainbow, Richard Eccles, Charlotte Nelson, Mark Whitehead, Lance Turtle, Sam T. Haldenby, Richard Gregory, Matthew Gemmell, Dominic Kwiatkowski, Thushan I. de Silva, Nikki Smith, Adrienn Angyal, Benjamin B. Lindsey, Danielle C. Groves, Luke R. Green, Dennis Wang, Timothy M. Freeman, Matthew D. Parker, Alexander J. Keeley, Paul J. Parsons, Rachel M. Tucker, Rebecca Brown, Matthew Wyles, Chrystala Constantinidou, Meera Unnikrishnan, Sascha Ott, Jeffrey K.J. Cheng, Hannah E. Bridgewater, Lucy R. Frost, Grace Taylor-Joyce, Richard Stark, Laura Baxter, Mohammad T. Alam, Paul E. Brown, Patrick C. McClure, Joseph G. Chappell, Theocharis Tsoleridis, Jonathan Ball, Dimitris Gramatopoulos, David Buck, John A. Todd, Angie Green, Amy Trebes, George MacIntyre-Cockett, Mariateresa de Cesare, Cordelia Langford, Alex Alderton, Roberto Amato, Sonia Goncalves, David K. Jackson, Ian Johnston, John Sillitoe, Steve Palmer, Mara Lawniczak, Matt Berriman, John Danesh, Rich Livett, Lesley Shirley, Ben Farr, Mike Quail, Scott Thurston, Naomi Park, Emma Betteridge, Danni Weldon, Scott Goodwin, Rachel Nelson, Charlotte Beaver, Laura Letchford, David A. Jackson, Luke Foulser, Liz McMinn, Liam Prestwood, Sally Kay, Leanne Kane, Matthew J. Dorman, Inigo Martincorena, Christoph Puethe, Jon-Paul Keatley, Gerry Tonkin-Hill, Christen Smith, Dorota Jamrozy, Mathew A. Beale, Minal Patel, Cristina Ariani, Michael Spencer-Chapman, Eleanor Drury, Stephanie Lo, Shavanthi Rajatileka, Carol Scott, Keith James, Sarah K. Buddenborg, Duncan J. Berger, Gaurang Patel, Maria V. Garcia-Casado, Thomas Dibling, Samantha McGuigan, Hazel A. Rogers, Adam D. Hunter, Emily Souster, Alexandra S. Neaverson, Ian Goodfellow, Nicholas J. Loman, Oliver G. Pybus, David L. Robertson, Emma C. Thomson, Andrew Rambaut, Thomas R. Connor

**Affiliations:** 1 MRC Centre for Global Infectious Disease Analysis, School of Public Health, Imperial College London, London, UK; 2 Institute of Evolutionary Biology, University of Edinburgh, Edinburgh, UK; 3 School of Biosciences, Cardiff University, Cardiff, UK; 4 Pathogen Genomics Unit, Public Health Wales NHS Trust, Cardiff, UK; 5 Institute of Microbiology and Infection, University of Birmingham, Birmingham, UK; 6 MRC-University of Glasgow Centre for Virus Research, Glasgow, UK; 7 Institute of Biodiversity, Animal Health and Comparative Medicine, Boyd Orr Centre for Population and Ecosystem Health, University of Glasgow, Glasgow, UK; 8 https://www.cogconsortium.uk/; 9 Department of Pathology, University of Cambridge, Cambridge, UK; 10 Department of Zoology, University of Oxford, Oxford, UK; 11 Department of Pathobiology and Population Sciences, The Royal Veterinary College, London, UK; 12 Quadram Institute Bioscience, Norwich, UK

**Keywords:** COVID-19, SARS-CoV-2, evolution, founder effect, epidemiology, spike

## Abstract

Global dispersal and increasing frequency of the SARS-CoV-2 spike protein variant D614G are suggestive of a selective advantage but may also be due to a random founder effect. We investigate the hypothesis for positive selection of spike D614G in the United Kingdom using more than 25,000 whole genome SARS-CoV-2 sequences. Despite the availability of a large dataset, well represented by both spike 614 variants, not all approaches showed a conclusive signal of positive selection. Population genetic analysis indicates that 614G increases in frequency relative to 614D in a manner consistent with a selective advantage. We do not find any indication that patients infected with the spike 614G variant have higher COVID-19 mortality or clinical severity, but 614G is associated with higher viral load and younger age of patients. Significant differences in growth and size of 614G phylogenetic clusters indicate a need for continued study of this variant.

## Highlights

• Increasing frequency of SARS-CoV-2 D614G is consistent with a selective advantage
• Phylodynamic analyses do not show significantly different growth of D614G clusters
• There is no association of D614G replacement with greater severity of infection
• The D614G replacement is associated with higher viral loads and younger patient age

## Graphical Abstract

**Figure:**
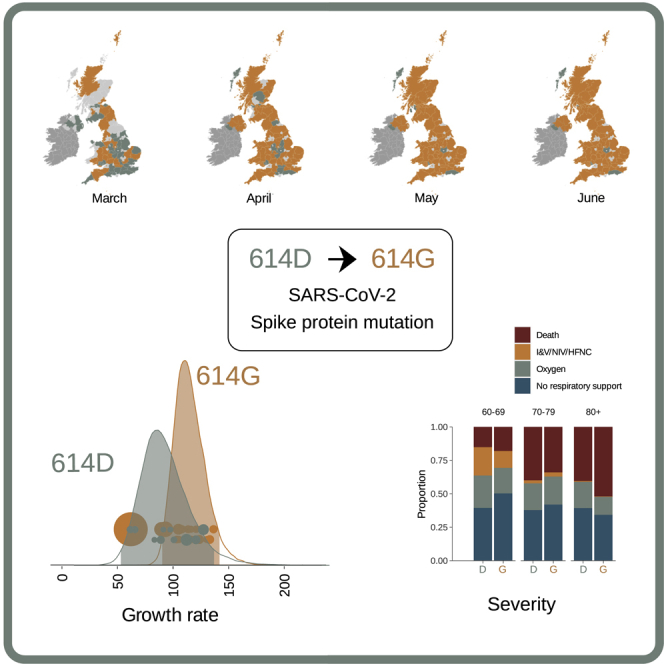

## Teaser

Analysis of the spread and frequency of SARS-CoV-2 D614G in the United Kingdom suggests a selective advantage for this strain that is associated with higher viral loads in younger patients but not higher COVID-19 clinical severity or mortality.

## Introduction

Severe acute respiratory syndrome coronavirus 2 (SARS-CoV-2), the coronavirus causing the global COVID-19 pandemic, is a rapidly evolving RNA virus that continually accrues genomic mutations as it transmits. A major focus of current research into SARS-CoV-2 genetics is whether any of these mutations have the potential to significantly alter important viral properties, such as the mode or rate of transmission, or the ability to cause disease. Evolutionary theory predicts that most new viral mutations are deleterious and short-lived, whereas mutations that persist and grow in observed frequency may be selectively neutral or advantageous to viral fitness. Discriminating between neutrality and positive selection is challenging, particularly for a newly emergent virus such as SARS-CoV-2. For example, the observation that a new mutation is increasing in prevalence or geographic range is, by itself, insufficient to prove its selective advantage to the virus because such increases can be generated by neutral epidemiological processes such as genetic bottlenecks following founder events and range expansions.

Considerable attention has focused on the D614G mutation in SARS-CoV-2, a non-synonymous mutation resulting in a replacement of aspartic acid with glycine at position 614 of the virus’s spike protein (D614G). The trimeric spike protein, composed of subunits S1 and S2, is a large glycoprotein that mediates cell entry and has been studied extensively in other coronaviruses, including SARS-CoV (Belouzard et al., 2009; Li, 2015; Li et al., 2005) and Midde East respiratory syndrome (MERS) (Millet and Whittaker, 2014; Yang et al., 2014). SARS-CoV-2 spike protein binds to angiotensin-converting enzyme 2 (ACE2) to gain cell entry, hence mutations in this gene have the potential to alter receptor binding affinity and infectivity, as well as viral immune evasion and immunogenicity (Watanabe et al., 2020).

The putative importance of the D614G mutation is based on three distinct sets of observations. First, experimental work using pseudotyped lentiviruses indicate that D614G increases infectivity *in vitro* (Korber et al., 2020; Yurkovetskiy et al., 2020; Zhang et al., 2020). Second, structural analysis suggests that D614G alters the receptor binding conformation, such that ACE2 binding and fusion is more likely (Yurkovetskiy et al., 2020). Third, analysis of the frequency of the 614D and 614G variants over time (based on submissions to global sequence databases) have suggested that locations that reported 614D viruses early in the pandemic were often later dominated by 614G viruses (Furuyama et al., 2020; Korber et al., 2020). More recent experimental work has contrasted spike 614 variants in animal models and human cell cultures using infectious cDNA clones of circulating SARS-CoV-2 strains. Enhanced replication in the upper respiratory tract (Plante et al., 2020) and enhanced transmission (Hou et al., 2020) of the 614G variant has been demonstrated in animal models of SARS-CoV-2 infection. Combined with epidemiological observations of disparities in viral loads in the upper respiratory tract (Lorenzo-Redondo et al., 2020; Wölfel et al., 2020), these results are suggestive of a transmission-mediated fitness differential between spike 614 variants.

**Figure 1:**
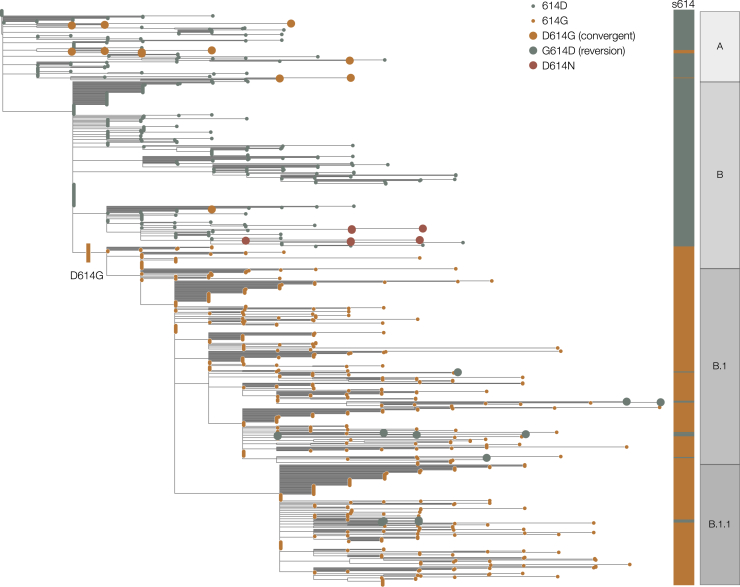
Maximum Likelihood Phylogeny Estimated from a Representative Set of 900 SARS-CoV-2 Genome Sequences, Showing global Lineage Assignments and the Origins of the Spike Protein D614G Mutation, which Seeded Many Introductions in the United Kingdom Putative reversions to 614D and independently arising D614G mutations are shown as large circles. The D614N genomes shown as red circles indicated two independent clusters in the United Kingdom.

**Figure S1:**
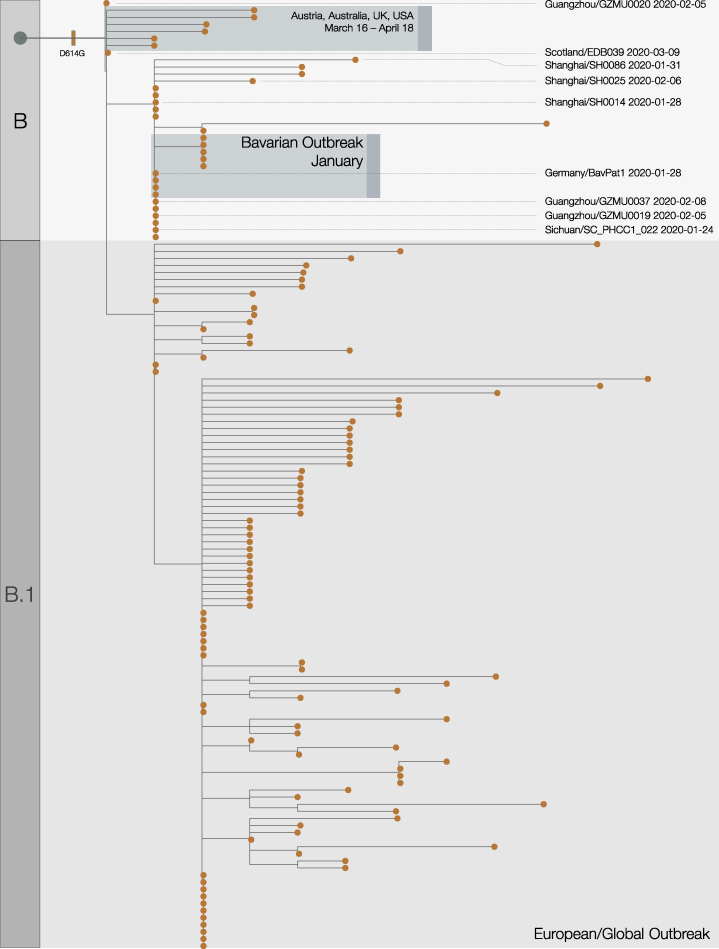
Expanded Phylogenetic Tree, Related to Figure 1 This shows the early stages of emergence of D614G into Europe from China. Acknowledgments and details for highlighted genome sequences are given in Table S3.

The D614G mutation is associated with the B.1 lineage of SARS-CoV-2 (Figure 1), which now dominates the global pandemic, based upon global SARS-CoV-2 genome sequences shared via GISAID (https://cov-lineages.org/lineages/lineage_B.1.html). Retrospectively sampled viruses suggest this mutation was present in Guangzhou, Sichuan, and Shanghai Provinces, China in late January (Figure S1). In Europe, the 614G variant was first observed in genomes sampled on January 28 in a small outbreak in Bavaria, Germany, which was initiated by a visitor from Shanghai (Rothe et al., 2020) and subsequently controlled through public health efforts. It is therefore likely that the D614G mutation occurred in China before being introduced on multiple occasions to European countries (Lai et al., 2020) where it increased in frequency. This scenario is consistent with the rapid increase in February and March of European virus genomes that carry the 614G variant (Dearlove et al., 2020; Korber et al., 2020). In the United Kingdom, the first observation of a genome carrying the D614G mutation was in a sample collected on February 28 from a patient in Scotland who had recently traveled through Italy (Robertson, 2020).

There is currently no scientific consensus on the effect of the D614G mutation on SARS-CoV-2 infectivity and transmissibility, and there is some skepticism that it could produce a meaningful effect at the population level given that SARS-CoV-2 is already highly transmissible and rapidly spreading (van Dorp et al., 2020; Grubaugh et al., 2020). The effect of the D614G replacement has been characterized *in vitro* with pseudotype virus and an *in vivo* in animal models, but this may not accurately recapitulate the effect of variants on virus transmissibility within the human population. Therefore, experimental evidence should be complemented with large-scale population studies that can detect meaningful changes in human-to-human transmission. The small size of the SARS-CoV-2 genomic datasets from many countries precludes robust analysis on a national scale. The substantially larger global SARS-CoV-2 sequence dataset is also problematic because of limited sequence metadata and variable sampling approaches among countries. To determine statistically if there is a meaningful difference in transmission between the 614D and 614G variants, we ideally need to observe repeated independent introductions of each variant into the same population and follow the trajectories of the outbreaks they cause.

In the United Kingdom, the rapid establishment of a national sequencing collaboration at the start of the epidemic, The COVID-19 Genomics UK consortium (COG-UK) (COG-UK, 2020), has resulted in the generation of >40,000 SARS-CoV-2 sequences from the country in <6 months (approximately half of all genomes sequenced globally as of the July 7). COG-UK has facilitated the usage of robust and systematic sampling and shared bioinformatic and laboratory approaches and the collection of consistent core metadata, resulting in a large, high-resolution dataset capable of examining changes in virus biology in the United Kingdom. Crucially for this study, and in contrast to epidemics that followed the first European outbreaks, the UK epidemic is the result of repeated introduction of SARS-CoV-2 from numerous global locations, including a substantial number of phylogenetic sub-trees (clusters) carrying either 614D and 614G. Here, we use the COG-UK dataset to examine evidence for increased transmissibility of SARS-CoV-2 due to genetic changes in its Spike protein. We also investigate the influence of spike 614D versus G on pathogenicity by matching sequence data with clinical outcome.

## Results

**Figure 2:**
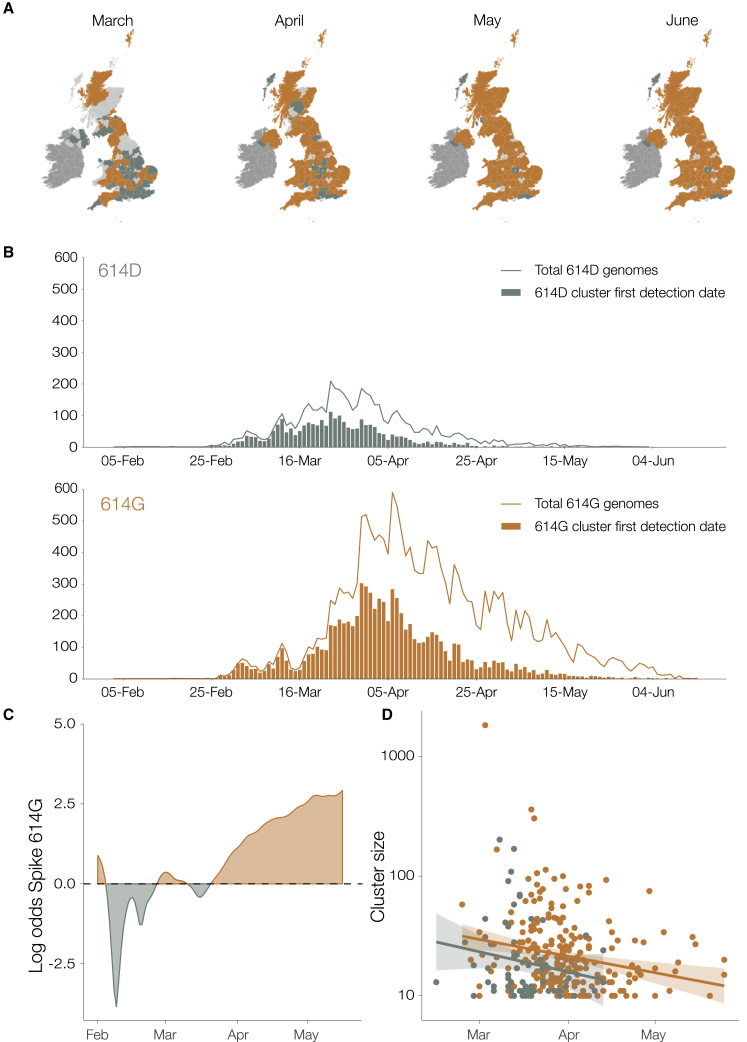
Geographic and Temporal Distribution of UK Phylogenetic Clusters, Classified as 614D or 614G According to the Residue They Carry at Spike Protein Position 614D (A) Shaded regions show the predominant residue in each region on the 15th of each month for March, April, May, and June 2020, with orange indicating that 614G was more frequently sampled and green indicating that 614D was more (or equally) frequent. Light gray indicates that no sequences had been sampled by that point in time. Dark gray indicates the Republic of Ireland. (B) The date when each cluster was first detected in the United Kingdom for variants 614D and 614G. Each cluster contains two or more sampled genomes. Solid lines show the total number of sequences collected by day of each 614 variant. (C) The log odds of sampling a 614G variant over time. (D) The size of cluster versus time of first sample collected within a cluster.

We identified 21,231 614G and 5,755 614D de-duplicated whole genome sequences sampled from different infections within the United Kingdom with known dates of sample collection between January 29 and June 16, 2020. We identified phylogenetic clusters of UK genomes using a maximum-parsimony reconstruction of the location of phylogenetic branches within the global SARS-CoV-2 phylogeny (see STAR Methods). Each cluster stems from one or a small number of introductions of the virus into the United Kingdom. We identify 245 614G and 62 614D clusters containing UK virus genomes from 10 or more different patients, after removing samples with spike 614 genotype, which does not match the majority within their cluster (reversions or contaminations). Importantly, we identified more UK phylogenetic clusters carrying the 614G variant than the 614D variant, and on average the 614G clusters were first detected later (the mean detection date for 614G clusters was 16 days later than of 614D clusters; Figure 2). While the frequency of sampling of 614G and 614D variants in the UK was close to parity in February and March, 614G became the dominant form in late March and this trend has continued (Figure 2C).

### Evaluating the Hypothesis that 614G Confers Increased Transmission Fitness

UK phylogenetic clusters that were first detected early in the epidemic tend to be larger than those detected later (Figure 2D). Although most 614G clusters tended to be detected later, they are on average 59% larger than 614D clusters after adjusting for the time of cluster detection (p = 0.008).

To evaluate if the increasing frequency of 614G reflects a selective advantage, we fit a logistic growth model to the chronological date that each specimen was sampled in the population under the assumption that sequences are sampled in proportion to the prevalence of each spike 614 variant. Under this model, 614D-infected cases grow exponentially at a rate r and 614G-infected cases grow exponentially at rate r(1+s), where s represents the estimated mutational selection coefficient.

In order to account for the rapid increase in SARS-CoV-2 introduction into the United Kingdom during March (du Plessis et al., 2020), we adapted the logistic growth model to count only those sequences that belong to clusters first detected in January or February. We further limit the analysis to sequences sampled during a period of exponential growth up to the end of March shortly after a national lockdown was implemented in the United Kingdom. Origin times for clusters were estimated using molecular clock phylogenetic methods (cf. STAR Methods). We also only consider samples collected after the most recent common ancestor (TMRCA) of the individual TMRCA of all clusters and where there are at least 10 samples with *either* amino acids 614D or G. Under these conditions, all samples included in the analysis were collected during a period when the selected clusters were co-circulating within the United Kingdom.

**Figure 3:**
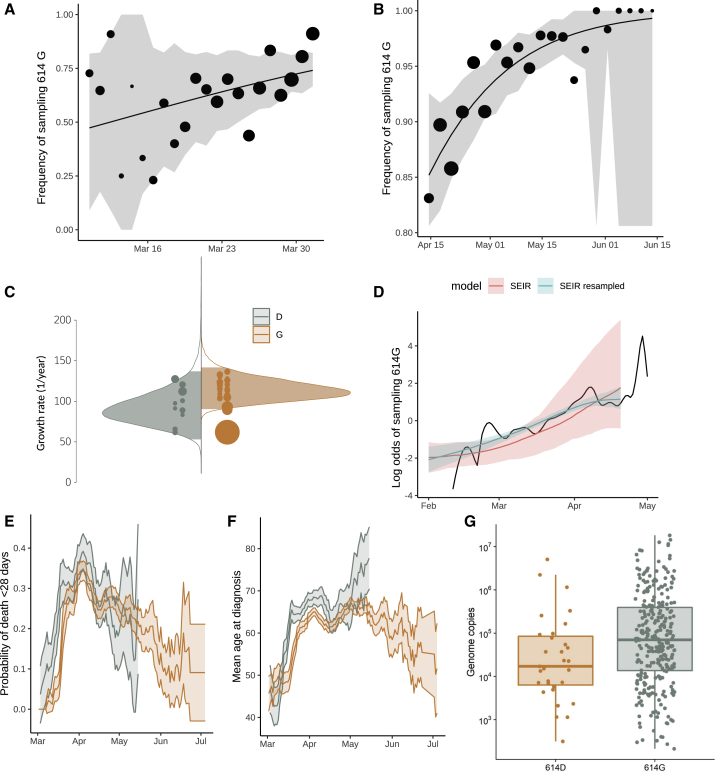
Relative Frequency of Spike 614D and G over Time, Phylodynamic Growth Rates and Comparison of Clinical Severity Metrics Relative frequency of spike 614D and G over time (A and B), phylodynamic growth rates (C and D), and comparison of clinical severity metrics (E–G). (A) Frequency of sampling spike 614G over time for clusters sampled during exponential growth phase. The size of points represents the number of samples collected on each day. The line and shaded region showed the maximum likelihood estimate (MLE) and confidence interval fit of the logistic growth model. (B) As in (A) but including samples during a period after April 15 during a period of epidemic decline. (C) Distribution of exponential growth rate for spike 614G (brown) and 614D (gray) in units of 1/year. Solid areas span the 95% credible interval. Points indicate the rates estimated for specific clusters and are sized by the number of sequences in that cluster. (D) Log odds of sampling spike 614G in London comparing empirical values (black line) and estimates based on the phylodynamic susceptible-exposed-infectious-recovered (SEIR) model (shaded regions). The green shaded region shows estimates making use of both genetic data and sample frequency data. (E) The probability over time of fatal outcome within 28 days of diagnosis among UK patients with sequence data that can be matched to clinical records. Shaded regions show 95% confidence region of a 7-day moving average. Points with fewer than 20 observations are omitted. (F) Moving average of age among samples included in (E). (G) Viral load (real-time qPCR mean genome copies) estimated using SARS-CoV-2 RNA strands from 31 614D (614D) and 290 614G samples.

**Figure S2:**
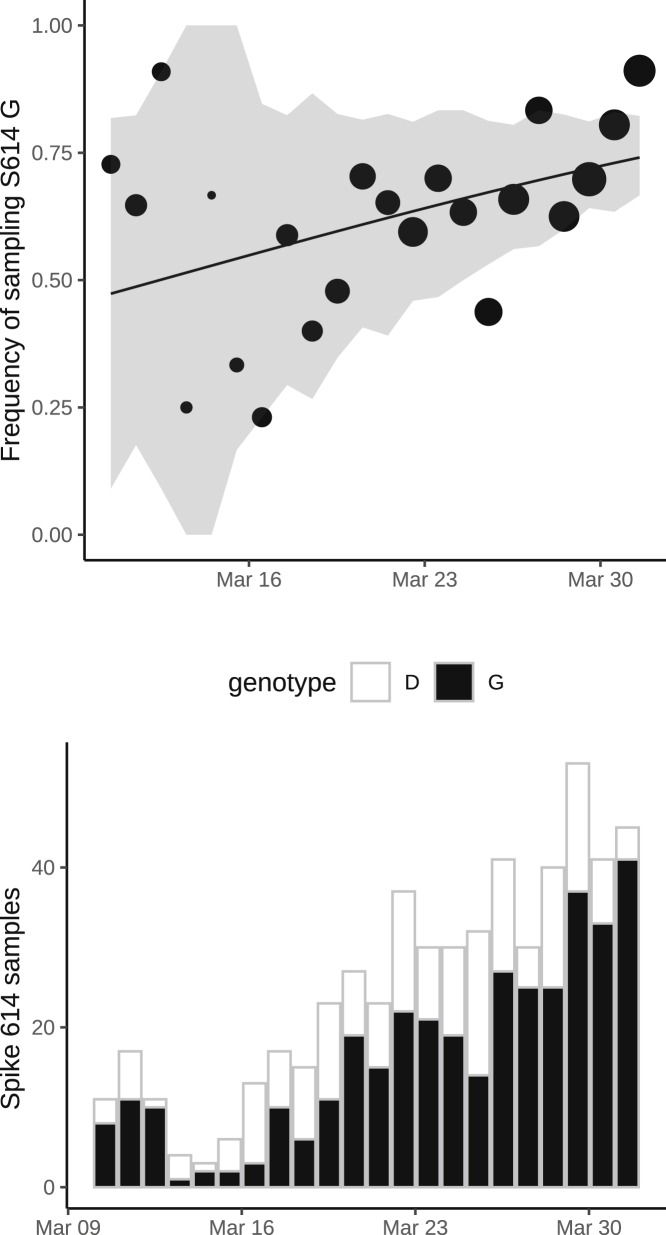
Frequency of Sampling Spike 614G over Time, Related to Figure 3 This shows frequency and numbers of Spike 614G and Spike 614D samples over time. The size of points represents the number of samples collected on each day. The line and shaded region showed the MLE and confidence interval fit of the logistic growth model.

Consequently, for this analysis, we retained five 614D clusters (n = 355 sequences) and five 614G clusters (n = 1,855 sequences) and estimated a selection coefficient for the 614G of 0.21 (95% CI: 0.06–0.56) (Table 1). The observed and fitted frequencies of 614G samples are shown in Figures 3A and S2. Information used to fit this model is drawn disproportionately from late March when more sequences are available.

**Table 1:** Estimates of the Selection Coefficient of the 614G Variant Using Different Datasets and Models

Method	Selection Coefficient
Logistic growth phase	0.21 (0.06, 0.56)^a^
Logistic decline phase	0.27 (0.12–0.54)^a^
“Boom-bust” coalescent model	0.29 (−0.24, 1.18)^b^
Skygrowth coalescent	0.17 (−0.24, 0.57)^b^
London SEIR structured coalescent	0.10 (−0.15, 0.41)^b^
London SEIR with sample frequency data	0.26 (−0.01, 0.58)^b^

a maximum likelihood estimate (95% confidence interval)

b median posterior (95% credible interval)

**Figure S3:**
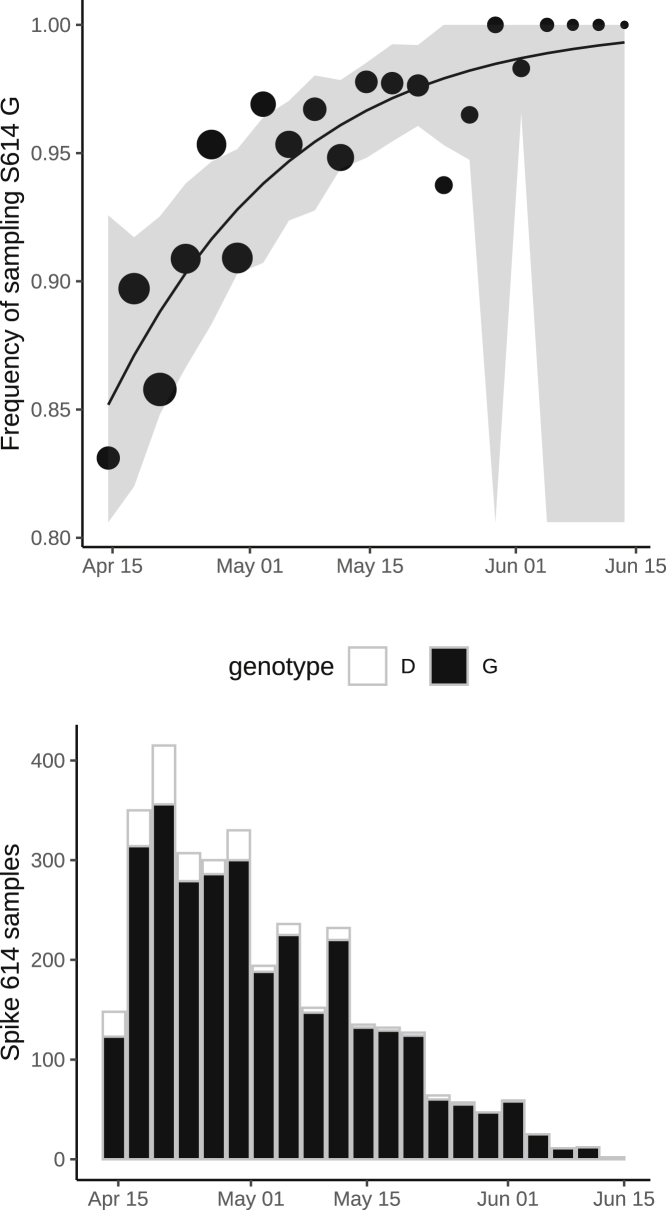
Frequency of Sampling Spike 614G after April 15, Related to Figure 3 This shows frequency and numbers of Spike 614G and Spike 614D samples over time using 37 DT clusters detected before March 31, 2020. The size of points represents the number of samples collected on each day. The line and shaded region showed the MLE and confidence interval fit of the logistic growth model.

We separately fitted the logistic growth model to the period of epidemic decline after April 15. If we include all clusters first detected before March 31, then we have n = 3,335 sequences (3,093 614G and 242 614D) sampled after April 15 and belonging to 37 phylogenetic clusters. This cross-section of data also exhibits increasing frequency of 614G through time (Figures 3B and S3), with an estimated selection coefficient of 0.27 (95% CI: 0.12–0.54).

An alternative source of information about the relative growth rates of the two variants comes from changing patterns of genetic diversity over time in each cluster. We applied phylodynamic methods (Pybus and Rambaut, 2009) to estimate effective population size and effective growth rates over time. First, we applied a parametric “boom-bust” exponential growth coalescent model to all clusters containing >40 samples, giving 50 clusters (11 for the 614D variant and 39 for 614G).

**Figure S4:**
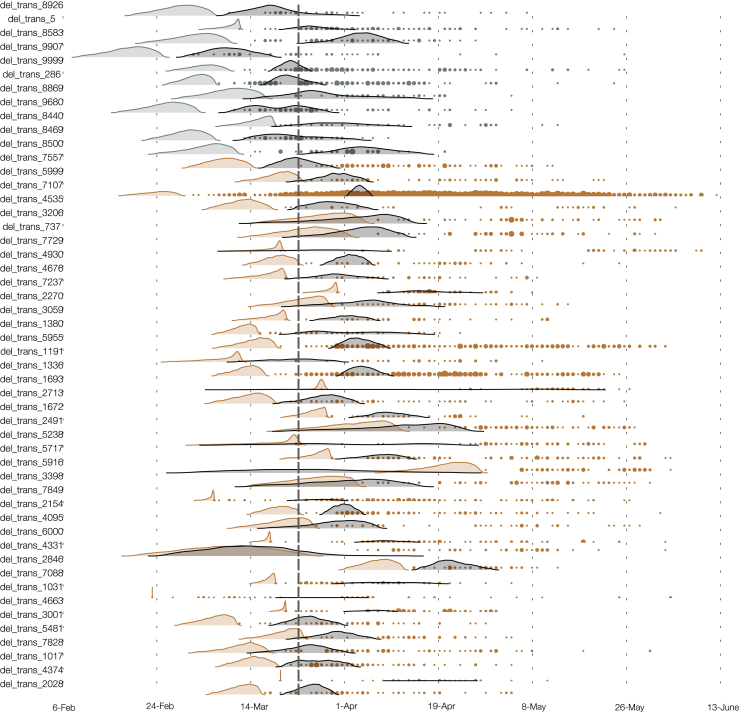
The Estimated TMRCA for Each of 50 UK Clusters (Shaded Density) and Time of Each Sequence Sampled (Points), Related to Figure 3 Brown and gray respectively indicate Spike 614G and 614D clusters.

**Figure S5:**
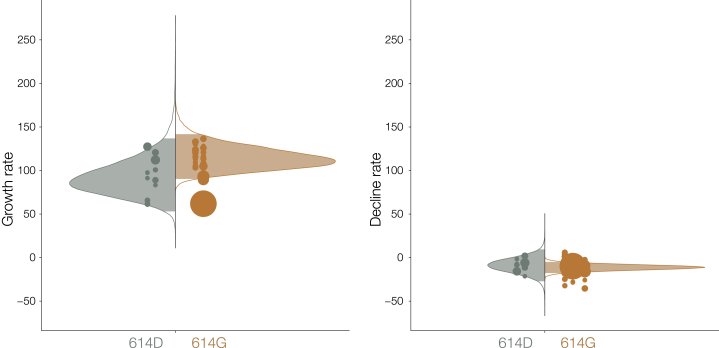
Distribution of Exponential Growth Rates (Left) and Rates of Decline (Right) for Spike 614G (Brown) and 614D (Gray) in Units of 1/Year, Related to Figure 3 Solid areas span the 95% credible interval. Points indicate the rates estimated for specific clusters, and are sized by the number of sequences in that cluster.

Under this model, population size grows exponentially up to a transition time, whereupon it shrinks exponentially. Rates of growth and decline and the transition time can vary for each 614G and 614D cluster, but a joint estimate for these are obtained using a hierarchical model (see STAR Methods). Among the 50 clusters, the 614G clusters tended to start later and persist longer than 614D (Figure S4), while 614D clusters tended to have slightly earlier transition times (614D mean = March 25, 614G mean = April 1). We do not detect any significant evidence for positive selection of the 614G variant using this model (Table 1), as uncertainty in estimated cluster growth rates was large (Figures 3 and S5). Growth rates for 614G clusters tended to be larger (posterior mean = 114 year^−1^, versus 93 year^−1^) as too were the decline rates of 614G clusters (posterior mean = −11 year^−1^, versus −9 year^−1^) but these differences were non-significant.

**Figure S6:**
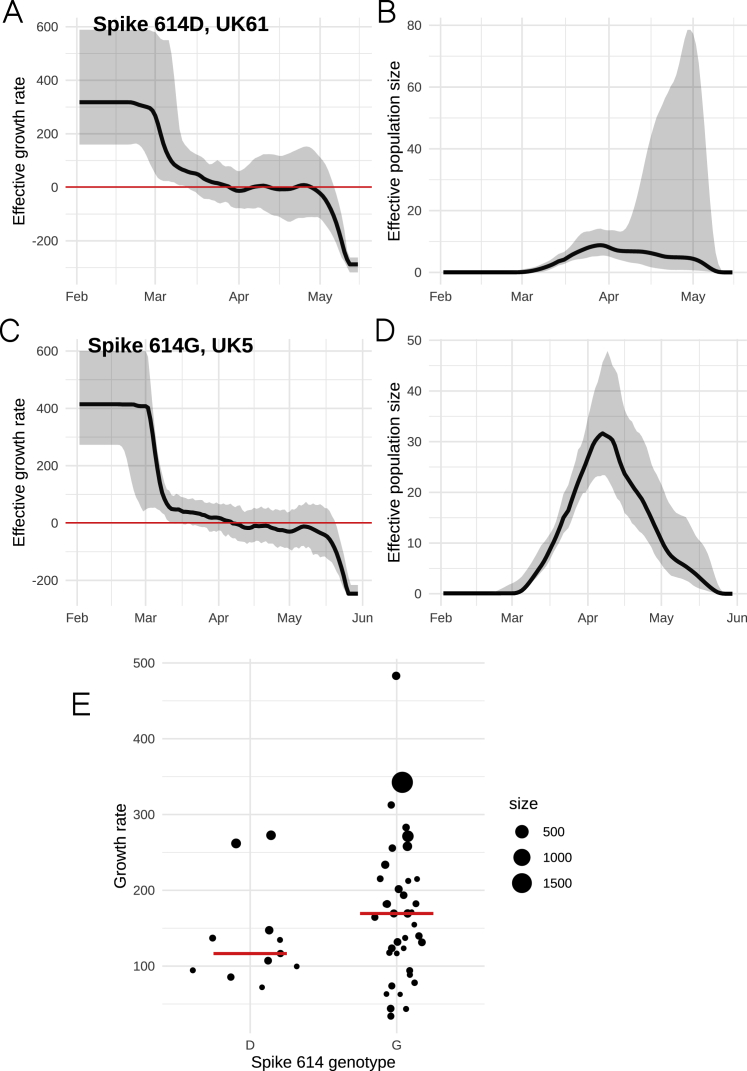
Non-parametric Phylodynamic Estimates for Representative Clusters, Related to Figure 3 Estimated growth rates (A and C) and effective population size (B and D) for the two largest clusters with genotypes Spike 614D/G. The growth rate at the beginning of the time axis (Feb 1, 2020) is shown in panel E and provides a data point for the statistical comparisons between clusters. The size of points corresponds to the number of samples in each cluster.

Further, we applied a non-parametric phylodynamic model that allows virus population size growth rate to vary over time according to a stochastic process. We applied this model independently to each of the clusters described above. We found that effective population size in the largest clusters tracks the progression of the epidemic in the United Kingdom and growth in most clusters is negative by early April 2020 (Figures S6A–S6D). We then examined if the 614G variant explained variance in growth rates among phylogenetic clusters. The initial growth rate of each cluster was highly variable (Figure S6), and precision of the estimated rate was generally low. The spike protein 614 polymorphism on its own explains very little variance in growth rates among clusters (weighted least-squares R^2^ = 1%), and there was no significant difference in initial growth rates (median initial growth rate for 614D clusters = 117 year^−1^ versus 169 year^−1^ for 614G clusters; Kruskal Wallis p = 0.13). This corresponds to an R_0_ of 3.1 (interquartile range, IQR: 2.7–3.5) for 614D clusters and 4.0 (IQR: 3.1–4.8) for 614G clusters, assuming a 6.5 day serial interval (Flaxman et al., 2020). The region of sample collection was not significantly associated with growth rates (weighted least-squares, p = 0.248). We did not observe a significant association between growth rates and the first detection date of a cluster (weighted least-squares, p = 0.62).

We next examined if there was a detectable difference in growth rates by combining information from the virus phylogeny and the empirical frequency of sampling of the 614G variant over time. We conducted a model-based phylodynamic analysis using 200 sequences sampled randomly from the London metropolitan area (cf. STAR Methods). A phylogeographic model specified the relationship between the London sequences and a random sample of 100 sequences from outside of London, thereby providing a mechanism to control for founder effects. Figure 3D shows the estimated frequency of 614G and 614D infections over time in London using this approach. We estimated that 614D was initially the most prevalent variant but that 614G overtook 614D in late March. A similar transition from 614D to 614G was observed in the empirical sampling frequencies, such that by the end of March, samples from London are more than twice as likely to be the 614G variant. The phylogeographic model was fitted both with and without information about sampling frequency of 614G over time. Incorporating sampling information into the mode increases the estimated selection coefficient, from 0.10 (without sampling information) to 0.26 (95% CI: −0.01–0.58) (Table 1). It is important to note that all fitted trajectories predict that the log odds of sampling 614G increase even if the selection coefficient is zero and that this is not necessarily evidence of positive selection for 614G.

### Association of Spike 614 Replacement with Infection Severity, Outcome, and Age

**Figure 4:**
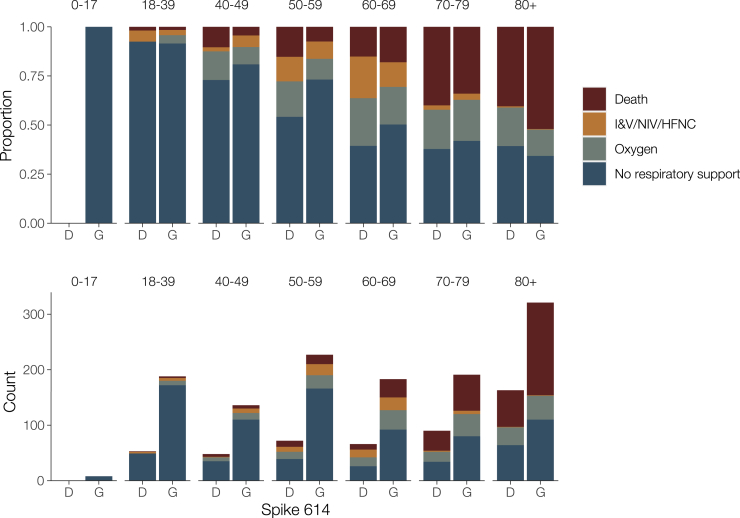
Clinical Severity in Patients in Association with the D614G Polymorphism and Age Clinical severity was measured on a four-point ordinal scale based on requirement for respiratory support. Upper panel: proportion of outcomes by age; lower panel: absolute counts. I&V, intubation and ventilation; NIV, non-invasive ventilation; HFNC, high-flow nasal cannulae; Oxygen, supplemental oxygen delivered by face mask or low-flow nasal cannulae.

We investigated associations between the D614G polymorphism and virulence by linking virus genome sequence data with clinical data on patient outcomes. We studied two clinical outcome datasets: dataset 1 – 9,782 614G- and 2,533 614D-associated genetic sequences collected by Public Health England between February 3 and July 4, 2020 linked to patient outcome after 28 days post-diagnosis (death or recovery), and dataset 2 – 1,670 (486 614D and 1,184 614G) genetic sequences collected by NHS Greater Glasgow and Clyde between February 28 and June 30, 2020 linked to records of clinical severity. In univariate analyses of dataset 1, we found that patients with the 614G variant show reduced odds of death, but this effect disappeared after controlling for other known risk factors for severe COVID-19 outcomes (Table 2). Mortality closely tracks average age within our sample, which varied greatly over time as testing priorities changed (Figures 3E and 3F). We observed associations between time of sampling (chronological date when specimens were collected) and genotype (later samples were more likely to have 614G) and later samples having higher odds of death and higher age. Odds of survival decrease for later samples, which may reflect prioritization of very severe cases for hospitalization and genetic sequencing as the epidemic peaked in March and April. For dataset 2, clinical severity was recorded using an ordinal scale based on oxygen requirement (1: no respiratory support, 2: supplemental oxygen, 3: invasive or non-invasive ventilation or high flow nasal cannulae, and 4: death). The association between the D614G polymorphism and severity of disease was estimated with high uncertainty, but the posterior was centered close to zero indicating that a biologically relevant effect is unlikely (mean: 0.03; 95% CI: −0.80–0.84). Increasing age and male biological sex were both associated with a marked increase in clinical severity (Figure 4; Table S1). We found a correlation in infection severity of patients with phylogenetic relatedness of the virus (mean standard deviation of the phylogenetic random effect: 0.26; 95% CI: 0.19–1.09). However, it is unclear to what extent this correlation represents genetic differences between viruses underlying infection outcomes as opposed to being an artifact of related viruses being spatially co-located and thus infecting individuals with similar characteristics.

**Table 2:** Odds Ratios (ORs) of Death within 28 Days Post Diagnosis

Predictor	OR	Adjusted OR	Coefficient
614 G	0.82 (0.74–0.90)	1.09 (0.97–1.23)	0.09 (-0.03–0.21)
Sex=Male		2.15 (1.95–2.36)	0.77 (0.67–0.86)
Age			1.63 (1.56–1.70)
Time of sampling			−5.6 (−6.68 – −4.62)

Continuous variables were scaled (*Z* score) before regressing. Coefficients are in standardized units (*Z* score). 95% confidence intervals are shown in parentheses. Time of sampling is the chronological date when the specimen was collected and is not relative to patient diagnosis or symptom onset.

**Figure S7:**
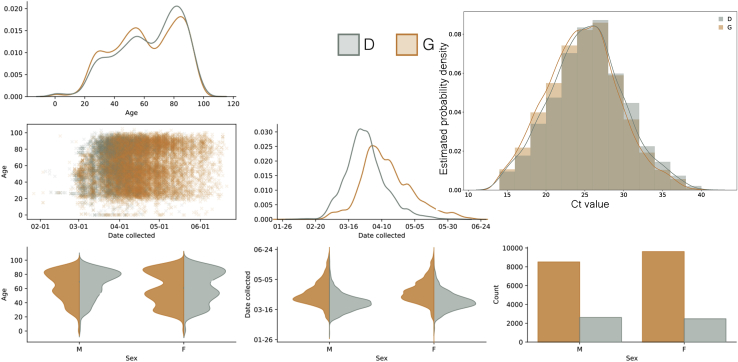
Probability of Observing Spike 614G Virus in Patients Grouped by Age and Sex, Related to Figure 4 Panels on the lower diagonal show collected pairwise plots based on a UK-wide (England, Wales, Scotland, and Northern Ireland) multivariate dataset for the sample collection date, and the age and sex of the patient. Kernel Density Estimation (KDE) and count plots are on the diagonal. The upper right panel shows the estimated frequency of PCR cycle threshold (Ct) for D/G variants overlaid with kernel density estimates. Samples where the amino acid at position 614 was not recorded and samples with a Ct value of less than 14 or greater than 40 were excluded.

We observed an association between age and genotype, with younger patients more likely to carry 614G viruses. We see this association despite the progressive aging of the patient cohort (Figure 3F) and concomitant increase in prevalence of 614G relative to 614D. We performed a multivariate analysis on the metadata of 27,038 sequences from across the United Kingdom (England, Wales, Scotland, and Northern Ireland) for the sample collection date and the age and sex of patients. A significant difference was found between the distribution of patient ages for 614G and 614D (Figure S7; Mann Whitney U test: p < 10^−13^). The median age is 5 years older among female carriers of 614D versus 614G and 4 years older among male carriers of 614D versus 614G. An association was also observed between sex and the presence of 614G or 614D (Figure S7; Chi-square test: p < 10^−10^). Differences in the age distribution for each sex were also observed (Mann-Whitney U p < 10^−8^ for 614D and p < 10^−37^ for 614G). The probability of carrying 614G virus seems to decrease continuously with age (Figure S7). This is possibly due to an increased viral load in younger patients associated with 614G variants leading to higher detection rates.

We observe a significant association between phylogenetic cluster membership and patient age, but this explains only 4.5% of variance in patient ages. We do not observe an association between the median patient age within clusters and cluster growth rates estimated using the non-parametric phylodynamic model (p = 0.13). Most phylogenetic clusters cover a very large range of patient ages. Of 32 clusters with at least 10 age observations, 31 clusters have an age range which spans values <35 to >85. Among 11 614D clusters, the median age ranges from 49 to 83, and among 39 614G clusters, the median age ranges from 42 to 85.

### Association of Spike 614 Replacement with Viral Load

As a proxy for viral load we studied 12,082 sequences with PCR cycle threshold (Ct) values from across the United Kingdom. Sequences with 14 ≤ Ct ≤ 40 were inspected for association with genotype, and a very slight (<1 Ct step) but significant difference was observed with 614G associated with lower Ct (Figure S6; p < 10^−6^, Mann-Whitney U test). As different test methods were used to obtain the Ct values across the dataset making a reliable comparison difficult, we carried out real-time quantitative viral load testing using a subset of 31 614D and 290 614G samples extracted on the same platform and analyzed using the 2019-nCoV_N1 assay real-time qPCR assay. This again found a significant difference with 614G associated with higher viral load (Figure 3G; p = 0.0151, Mann-Whitney U test).

### Other Proximal Residue Replacements with Potential Relevance to Spike Subunit Function Stability

Within the United Kingdom and global SARS-CoV-2 phylogenies, there are multiple instances of the D614G mutation as well as reversions back to 614D. The existence of reversions implies that the 614D variant is still relatively fit within individual hosts. Within the United Kingdom, we also observe two phylogenetic clusters of another variant, 614N, the independent origins of which suggest that this variant is also transmissible. However, the effect of 614N on spike subunit function remains to be determined.

We observed additional mutations at the residues immediately adjacent to spike 614. The mutation 613H co-occurs with both 614G and 614D, possibly as a result of convergent evolution. V615I occurs on the background of 614D, while V615F co-occurs with 614G (Table 3). These replacements are associated with one or two UK clusters, showing evidence for their transmission within the United Kingdom. Variant 615I is largely constrained to Wales, where it is associated with a large phylogenetic cluster that has not been observed since mid-April. In comparison to other polymorphic sites on the spike protein, the codons 613–615 appear to have moderately enriched diversity. Using an alignment of 55,653 UK sequences collected up to September 14, we find 107 sites on the spike protein with rare polymorphism of equal or greater prevalence than 613H and 615I. Considering these 107 sites, there are only four other regions on the spike protein with three consecutive polymorphisms (S26, S846, S1228, and S1252). Experimental studies will be required to determine whether these mutations proximal to site 614 have similar effects to 614G or, when co-occurring, have compensatory or epistatic effects.

**Table 3:** Circulating Amino Acid Haplotypes Found at Residues 613–615 of the SARS-CoV-2 Spike Protein

Haplotype Spike 613–615	Number of Observed Genomes	Number of UK Clusters	Date of First and Last Sample
QGF	3	2	2020-04-27, 2020-04-30
HDV	5	1	2020-03-30, 2020-03-30
QNV	7	2	2020-04-01, 2020-04-22
QDI	20	2	2020-03-17, 2020-04-15
HGV	24	3	2020-03-29, 2020-04-22
QDV	13356	2321	2020-01-29, 2020-06-03
QGV	43239	7007	2020-02-23, 2020-06-14

The ancestral haplotype is inferred to be QDV. The table reports the number of distinct UK clusters that the respective genomes are found in.

## Discussion

The spread of a virus mutation is governed by demographic processes such as population growth, range expansion, founder effects, and random genetic drift, as well as by potential positive selection if the mutation confers enhanced transmissibility. We used population-level data to epidemiologically evaluate the transmission fitness of the spike 614G by using a very large dataset of patient samples and a range of inference approaches. Not all methods show a conclusive signal of enhanced growth of the 614G variant. Given the many factors that contribute to transmission dynamics, it is unsurprising that the population-level values we have estimated are much less than the proportional increase in cell infectivity measured *in vitro* (Korber et al., 2020; Yurkovetskiy et al., 2020).

Estimating the epidemiological fitness of individual genetic variants during an emerging pandemic presents multiple challenges. The recent origin of SARS-CoV-2 combined with a relatively low rate of evolution means global viral genetic diversity is low, and many methods for identifying positive selection will have low sensitivity. Evidence for positive selection at spike position 614 and other sites has been suggested by statistical models based on the rate ratio of nonsynonymous to synonymous substitutions (Pond, 2020). However, the detection of positive selection by such methods does not necessarily imply the mutation enhances transmissibility, and effects of individual mutations on transmissibility will generally be low (MacLean et al., 2020).

Convergent molecular evolution (resulting in homoplasies) can present an alternative source of information about potentially beneficial virus mutations; however, such approaches lack sensitivity for the D614G as almost all circulating 614G genomes derive from a single ancestor (van Dorp et al., 2020). Our discovery of co-occurring mutations in neighboring sites (615 and 613) and the D614N variant is suggestive of a more complex selective landscape in this region of the spike protein than was first indicated. We also note that our analysis is limited by necessity to the comparison of co-circulating clusters that, in some cases, are characterized by mutations at sites other than 614; hence, it is impossible to disentangle the selective effects of each individual mutation. One amino acid replacement is notable: RdRp P323L, which occurred almost concurrently with D614G and is in almost perfect linkage equilibrium with 614G (Pond, 2020). The rarity of independent occurrences of D614G and P323L make it impossible to evaluate the effects of these replacements epidemiologically, but experiments with pseudotyped virus have been carried out in the absence of P323L.

We have drawn on two sources of information regarding the growth of the 614G variant: (1) the relative frequency of the 614G and 614D variants through time and (2) inferred differences in the genetic diversity and growth rate of 614G and 614D phylogenetic clusters in the United Kingdom (phylodynamics). While the changing frequency of one variant in an exponentially growing population can in theory indicate a difference in fitness, the rate at which 614G clusters were imported and discovered in the United Kingdom also changed through time, making direct comparisons of variant frequencies challenging. We controlled for this effect using phylogenetic analysis and by counting only samples derived from co-circulating clusters representing distinct introductions of SARS-CoV-2 into the United Kingdom. Separately, phylodynamic methods allow us to infer the growth and decline in effective population size of individual phylogenetic clusters, and we used this approach to compare the mean growth rates of 614G and 614D clusters. These phylodynamic estimates have high statistical uncertainty and do not consistently detect a significant difference in growth rate. We observed, however, that 614G clusters tend to grow to a larger size than 614D clusters after controlling for time of introduction into the United Kingdom. This is consistent with a transmission advantage of 614G variants but could also be the result of unknown confounders that increase the probability that 614G lineages will be sampled. Our data will naturally be biased toward samples that are easy to sequence, and we have observed a significant decrease of real-time PCR Ct values of the 614G variant, although the difference is very small. We may also observe larger 614G clusters if such clusters arise from importation of multiple genetically identical lineages and if this multiplicity is greater for 614G than 614D. This could, for example, occur due to greater transportation links with other European countries where 614G was rapidly expanding in March. We have not, however, found evidence for such an effect, and while this may partially explain larger 614G cluster sizes, such importation patterns would not bias frequency-based inference of selection coefficients that draws information from changes in genotype frequencies rather than initial conditions.

Phylodynamic estimates of reproduction numbers are sensitive to the context of early spread of epidemic clusters, which may have involved superspreading events (Endo et al., 2020). Such events can add variance to estimates of cluster-level reproduction numbers, which are already imprecise when based on poorly resolved phylogenies. Reproduction numbers based on phylogenetic clusters may not be representative of the epidemic as a whole and may be larger on average since they reflect lineage importations that were highly successful. Reassuringly, recent phylodynamic analysis of SARS-CoV-2 sequences by Miller et al. (2020) has shown that estimates of reproduction numbers are relatively insensitive to assumptions about superspreading events; however, estimates of epidemic size are highly dependent on superspreading events.

The observed association of patient age with D614G remains an unexplained and potentially important aspect of the epidemiology of this variant. Contact surveys have demonstrated decreasing rates of contact after the age of 40, which is suggestive of lower transmission rates in older age groups (Walker et al., 2020). If 614G is more prevalent in younger age groups, this may partially explain higher growth rates of this variant. But the mean age difference of 4–5 years is unlikely to correspond to a large difference in contact rates. We further show that phylogenetic clusters generally span a very large range of ages implying relatively rapid mixing between age groups. And the age difference between variants persists over the epidemic curve, long after most lineage importation events have occurred, indicating that the age difference is not a consequence of different initial conditions in spike 614G and 614D clusters.

SARS-CoV-2 case and infection fatality rates seem to vary widely among countries and through time. It is unclear to what degree this variation reflects estimation uncertainty, host population factors (such as the age structure of the population; Onder et al., 2020), or virus genetic factors. Here, we do not detect a difference in virulence between the two spike 614 variants. By estimating mortality rates as opposed to rates of hospitalization or ICU care, our results complement those in Korber et al., (2020) and are based on a substantially greater sample size. In addition, we did not find any association with clinical severity indicated by the requirement for oxygenation or respiratory support in a subset of 1,670 patients. A significant association of 614G carriage with age may indicate minor differences in clinical outcome or frequency of symptomatic infection, which bears further study. The data are heavily skewed toward hospitalized cases, and therefore more severe disease, and so it is not possible to evaluate small differences in virulence that may be present in milder or asymptomatic infections. This is especially problematic for evaluating effects that may be confounded by age, as the proportion of infections that do not lead to symptoms is higher in younger individuals (Davies et al., 2020).

Our analysis emphasizes that while laboratory experiments can identify changes in virus biology, their extrapolation to identify population level effects on transmission requires caution.

In the case of D614G, a large increase in cellular infectivity results in a weak population-level signal that nonetheless produces a discernible effect on transmissibility. While we believe an effect on SARS-CoV-2 transmissibility caused by D614G is likely to be present, it is important to note that the estimation of the absolute size of this effect is uncertain and much harder to predict. Although the signal is difficult to detect, the unprecedented size and completeness of the UK dataset and associated metadata enable many potential biases within the data to be controlled for. This work is therefore demonstrative of the value of large-scale coordinated sequencing activities to understand a pandemic in real time.

### Limitations of Study

Several limitations of the data and analysis should be considered when interpreting our findings. We applied classic population genetic models premised on contrasting the exponential growth rates of the 614G and 614D populations while controlling for founder effects, but in reality, the SARS-CoV-2 epidemic is noisy and structured in ways not accounted for by this model. The frequency of 614G and 614D variants can change rapidly due to stochastic fluctuations, especially early in the epidemic. The sampling process is also inhomogeneous through time and sometimes reactive to short-term public health situations (e.g., nosocomial outbreaks) rather than being fully randomized and systematic. Most of the SARS-CoV-2 genome sequencing performed by centers in the United Kingdom is focused on symptomatic cases, often using diagnostic residual samples. As testing priorities change, and as cases in different segments of the population fluctuate, signals may emerge that are due to operational changes rather than shifts in virus biology. This study shows that transmissibility of SARS-CoV-2 can change as the pandemic unfolds. Whether the current explosive epidemics across the world are to any degree being driven by D614G, or whether it is simply the beneficiary of being in the right place at the right time, it is now the dominant variant. Changes in the transmissibility of a circulating virus could have a major effect on pandemic planning and the effectiveness of pandemic response, and so it is critical that the parameters for models used for planning are based on the currently circulating virus. Work on vaccines, therapeutics, and other interventions must allow for this but also keep in mind that reversions, and other mutations at the same or adjacent residues, will undoubtedly emerge in the future.

## STAR★Methods

### Key Resources Table

**Table:** 

REAGENT or RESOURCE	SOURCE	IDENTIFIER
**Critical Commercial Assays**		

2019-nCoV_N1 assay RT-qPCR assay	(FDA https://www.fda.gov/media/134922/download)	Cat # 2019-nCoVEUA-01
NEB Luna Universal Probe One-Step Reaction Mix and Enzyme Mix	(New England Biolabs, Herts, UK)	Cat # E3006S
Applied Biosystems™ 7500 Fast PCR instrument running SDS software v2.3	(ThermoFisher Scientific)	Cat # 4351106

**Deposited Data**		

GISAID	(Shu and McCauley, 2017)	https://www.gisaid.org
European Bioinformatics Institute	(Rodriguez-Tomé and Stoehr, 1996)	https://www.ebi.ac.uk/
Issues with SARS CoV-2 Sequencing Data	(De Maio et al.).	https://github.com/W-L/ProblematicSites_SARS-CoV2/blob/master/problematic_sites_sarsCov2.vcf
A dynamic nomenclature proposal for SARS-CoV-2 lineages to assist genomic epidemiology	(Rambaut et al., 2020)	https://cov-lineages.org/lineages/

**Oligonucleotides**		

ARTIC V3 primers	https://github.com/joshquick/artic-ncov2019/blob/master/primer_schemes/nCoV-2019/V3/nCoV-2019.tsv	Available from IDT: https://eu.idtdna.com/pages/landing/coronavirus-research-reagents/ngs-assays

**Software and Algorithms**		

R 3.6.3	The R Foundation for Statistical Computing	http://www.R-project.org
BEAST1 v1.10.5	(Suchard et al., 2018)	https://beast.community/
Tracer	(Rambaut et al., 2018)	https://beast.community/
BEAST2 PhyDyn	(Bouckaert et al., 2019; Volz and Siveroni, 2018)	https://github.com/mrc-ide/PhyDyn
ARTIC network protocol	ARTIC network	https://artic.network/ncov-2019
R packages (*treedater* 0.5.1, *ape* package v. 5.3, *brms* v. 2.13.5, *rstan* v. 2.21.2, *SPIn* v. 1.1, *skygrowth* 0.3.1)	(Paradis and Schliep, 2019; Volz and Frost, 2017; Bürkner, 2018; Stan Development Team, 2020; Liu et al., 2015; Volz and Didelot, 2018)	http://www.R-project.org; https://github.com/mrc-ide/skygrowth
IQtree 1.6.12	(Minh et al., 2020; Rambaut et al., 2020)	http://www.iqtree.org/
MRC-CLIMB	(Connor et al., 2016)	https://www.climb.ac.uk/
Nextflow pipeline for processing/assembly of ARTIC protocol amplicons	https://github.com/connor-lab/ncov2019-artic-nf	https://github.com/connor-lab/ncov2019-artic-nf

### Resource Availability

#### Lead Contact

Further information and requests for resources and reagents should be directed to and will be fulfilled by the Lead Contact, Erik Volz (e.volz@imperial.ac.uk).

#### Materials Availability

This study did not generate new unique reagents.

#### Data and Code Availability

Genetic sequence data and limited metadata (sample collection date and country of origin) is available on GISAID (https://www.gisaid.org) and the Genomics UK Consortium (https://www.cogconsortium.uk/data/) which includes precomputed alignments and phylogenetic trees. Code to reproduce individual analyses are made available on GitHub.

### Experimental Model and Subject Details

#### Sample collection and sequencing

We utilized data from the Coronavirus Disease 2019 (COVID-19) Genomics UK Consortium (CoG-UK)( COG-UK, 2020), a partnership of more than 18 academic, medical and public health research centers contributing sequencing and analysis capabilities. Sequence data was generated from a variety of protocols and platforms and were uploaded to a centralized environment for storage and analysis (MRC-CLIMB) (https://www.climb.ac.uk/)(Connor et al., 2016). Data are uploaded with a standard set of clinical and demographic metadata and information about sequencing protocols and sample collection methods. Data undergo quality control and assembly and lineage assignment (Rambaut et al., 2020). Data which complete quality control and assembly steps are released on a weekly basis. Sequence data are periodically shared through two open access databases, the European Bioinformatics Institute(Rodriguez-Tomé and Stoehr, 1996) and the Global Initiative on Sharing All Influenza Data(Shu and McCauley, 2017). We utilized 26,986 whole genome sequences contained in the June 19 release (https://www.cogconsortium.uk/data/) and for which the Spike 614 genotype could be determined and sample collection date was known.

### Method Details

#### Phylogenetics and identifying clusters

Maximum likelihood (ML) phylogenetic trees were estimated separately using IQTree *v1.6.12* for major global lineages (Minh et al., 2020; Rambaut et al., 2020). Phylogenies were rooted on a sample from the ancestral lineage. UK clusters were identified using parsimony-based ancestral state reconstruction (Fitch, 1977) with internal nodes classified as UK or non-UK. Most UK clusters are descended from polytomies with descendents in multiple countries, and reconstruction of ancestral states at such nodes is ambiguous. In such cases the polytomy node was assigned the same state as it’s ancestor. We consider two extremes of the maximum parsimony method for reconstructing ancestral states at bifurcating nodes: We computed delayed transition (DT) parsimony assignments to each node which favors transition to the UK as far from the root as possible.

#### Parametric phylodynamic analysis

We used a two-epoch coalescent model to estimate a period of exponential growth followed by an independently estimated period of exponential decline. Note that although we refer to growth and decline, the growth rates for both epochs can take either positive or negative values. The transition time from growth to decline was estimated independently for each cluster using a normal prior with a mean of the 23rd March 2020 (2020.2254), the date of ‘lockdown’ in the UK, and a standard deviation of two weeks. The data consisted of delayed transition clusters of more than 40 sequences as of the 19th June 2020.

A normal hyperprior is specified for cluster growth/decline rates for each genotype and the mean and precision of the hyperprior are estimated. The posterior mean growth/decline rates for each genotype are estimated along with the growth/decline rate for each cluster individually. Posterior growth rates within each genotype are therefore correlated. The prior for the mean growth rate is Normal(0,100/year) and the prior of the precision parameter is Gamma(1,0.001). We compute the selection coefficient from growth rates with the formula $s=\left(r_{G}/r_{D}\right)-1$ where is the mean growth rate for each group of clusters.

The model was implemented in BEAST v1.10.5 (Suchard et al., 2018). Four independent chains of 100 m states were run for each variant, with 10% removed from each chain to account for burn-in. Convergence was assessed using Tracer(Rambaut et al., 2018) prior to further analysis. The HKY model was used to model nucleotide evolution (Hasegawa et al., 1985), and, following Duchene et al.(Duchene et al., 2020), the evolutionary clock rate was fixed at 0.001 substitutions per site per year. Other priors used are described in table S2. Code to reproduce this analysis can be found at https://github.com/COG-UK/D614G_spike_mutation_analysis (https://doi.org/10.5281/zenodo.4095529).

#### Non-parametric phylodynamic analysis

Rooted and dated phylogenies were estimated by randomly resolving polytomies in the ML trees described above using *ape 5.3(**Paradis et al., 2004**)* and *treedater* 0.5.1(Volz and Frost, 2017). The mean clock rate of evolution was constrained to (0.00075,0.0015). Branch lengths were smoothed by enforcing a minimum number of substitutions per site on each branch and by sampling from the distribution estimated by *treedater*. This was carried out 20 times for each UK lineage. Growth rates were estimated using *skygrowth* 0.3.1(Volz and Didelot, 2018) using Markov chain Monte Carlo (MCMC) and 1 million iterations for each time tree and using an Exponential(10^−4^) prior for the smoothing parameter. The final results were produced by averaging across 20 time trees estimated for each cluster. Code to reproduce this analysis is available at https://git.io/JJkIM and an interactive dashboard showing growth and decline of UK lineages can be viewed at https://shiny.dide.imperial.ac.uk/s614LineagesUK/.

#### Model-based phylodynamic analysis

We applied a susceptible-exposed-infectious-recovered (SEIR) model(Diekmann and Heesterbeek, 2000) for the SARS-CoV-2 epidemic in London linked to an international reservoir. The SEIR model assumed a 6.5 day serial interval. The estimated parameters included the initial number infected, the susceptible population size, and the reproduction number. The model included bidirectional migration to the region outside of London (both within the UK and internationally) at a constant rate per lineage. Evolution outside of London was modeled using an exponential growth coalescent. Additional estimated parameters include the migration rate, and the size and rate parameters for the exponential growth coalescent. This model was implemented in the BEAST2 PhyDyn package (Bouckaert et al., 2019; Volz and Siveroni, 2018) and is available at https://git.io/JJUZv. The phylogenetic tree was co-estimated with epidemiological parameters. In order to make results comparable between 614D and 614G lineages, the molecular clock rate of evolution was fixed at a value estimated using all data in *treedater* 0.5.1. Nucleotide evolution was modeled as a strict clock HKY process (Hasegawa et al., 1985). To fit the model we ran 20 MCMC chains for 20 million iterations, each using 4 coupled MCMC chains (Müller and Bouckaert, 2020). Bespoke algorithms were used to exclude chains which failed to sample the target posterior. We used identical uninformative Lognormal(mean log = 0, SD log = 1) priors for the reproduction number in 614G and 614D lineages.

The model was fitted to 614G and 614D sequence data separately before being combined for joint inference with the sample frequency data. This is carried out using a sampling-importance-resampling strategy(Smith and Gelfand, 1992). We sampled parameters from the posterior estimated from genetic data uniformly and computed importance weights using a sequential Bernoulli likelihood based on the estimated frequency of 614G and 614D over time. Parameters resampled 1 million times with these weights yield our final estimate of the posterior.

The selection coefficient given a ratio of reproduction numbers is computed as follows:

$$s=\frac{R_{0}^{G}}{R_{0}^{D}}-1$$

#### Clinical sample quantitative PCR

All samples were tested in duplicate using the 2019-nCoV_N1 assay RT-qPCR assay (https://www.fda.gov/media/134922/download); primers and probe were obtained ready-mixed from IDT (Leuven, Belgium). PCRs were performed in a final volume of 20 μl and included NEB Luna Universal Probe One-Step Reaction Mix and Enzyme Mix (New England Biolabs, Herts, UK), primers and probe at 500 nM and 127.5 nM, respectively, and 5 μl of RNA sample. No template controls were included after every seventh sample. Six ten-fold dilutions of SARS-CoV-2 RNA standards were tested in duplicate in each assay; standards were calibrated using a plasmid containing the N sequence that had been quantified using droplet digital PCR. Thermal cycling was performed on an Applied Biosystems 7500 Fast PCR instrument running SDS software v2.3 (ThermoFisher Scientific) using the following conditions: 55oC for 10 min and 95oC for 1 min followed by 45 cycles of 95oC for 10 s and 58oC for 1 min. Assays were repeated if the reaction efficiency was < 90% or the R2 value of the standard curve was £0.998. Where possible, testing of samples was repeated if the %CV of the duplicates was < 10%. Three samples were not tested in duplicate because of insufficient RNA. Two samples had Cq values that were below the top SARS-CoV-2 RNA standard in the assay. Duplicate PCRs from four samples had %CVs > 10 (range 10.19 to 17.06).

### Quantitative and Statistical Analysis

#### Statistical analyses

Size of clusters was evaluated using log-linear multivariate regression. Effect of genotype on phylodynamic growth rates was estimated using multivariate weighted regression. Regression weights are inversely proportional to precision of estimated growth rates. Univariate comparisons used the Kruskal Wallis test. Kernel density estimation of sample time distributions used Gaussian kernels and a bandwidth of 2 days. Statistical models were implemented in R 3.6.3.

#### Logistic growth model

According to this model the number of infected with the Spike 614D variant grows exponentially at a rate r and the number with the Spike 614G variant grows exponential at rate r(1+s). If $N_{X}$ is the number infected initially with variant X, the proportion of the population with Spike 614G at time t is

$$f_{G}\left(t\right)=\frac{N_{G}\;\exp\left(r\left(1+s\right)t\right)}{N_{G}\;\exp\left(r\left(1+s\right)t\right)+N_{D}\;\exp\left(rt\right)}$$

This model can be fitted to a sequence of sample times $\left(t_{1},\cdots ,t_{n}\right)$ with Spike 614 genotypes $\left(y_{1},\cdots ,y_{n}\right)$ by maximum likelihood. The objective function is

$$L\left(r,s,f_{G}\left(t_{0}\right)\right)=\sum_{i=1}^{n}I\left(y_{i}=G\right)\log\left(f_{G}\left(t_{i}\right)\right)+\left(1-I\left(y_{i}=G\right)\log\left(f_{G}\left(t_{i}\right)\right)\right)$$

Formally, fitting this model is equivalent to logistic regression of genotype on time where the coefficient corresponds to the compound parameter ρ=r×s. Deriving the selection coefficient therefore requires additional information about the growth rate r. For the model fitted to data during the exponential growth phase, we considered a range of plausible values for this rate corresponding to a reproduction number in the range 2.0-3.5 and a serial interval of 6.5 days(Flaxman et al., 2020). For the model fitted to data during the decline phase, we considered a rate corresponding to a generation time between 3 and 8 days. The final confidence interval is based on these ranges as well as the confidence interval of ρ computed using profile likelihood.

#### Analysis of severity of patient outcomes

We aggregated data from 1670 patients presenting with COVID-19 from NHS records and combined it with the genome sequence of the virus infecting them. We used a phylogenetic generalized additive model to investigate the viral D614G polymorphism and association with severity of the infection.

To control for the effect of other mutations in the genome, we generated a time tree of the virus genomes from Scotland using an HKY + Γ nucleotide model excluding the nucleotide position underlying the D614G mutation. We estimated the tree using IQ-TREE 2 v. 2.0.6 (Minh et al., 2020). We masked the nucleotide causing the D614G mutation, as well as all mutations recommended by De Maio et al. as of 22/7/2020 (https://virological.org/t/issues-with-sars-cov-2-sequencing-data/473/13). We included the first sequenced genome of SARS-CoV-2 from China (Wu et al., 2020) as an outgroup to root the tree.

We coded the severity of infection as four levels: 1) No respiratory support, 2) Supplemental oxygen, 3) Invasive or non-invasive ventilation or high flow nasal cannulae, 4) Death. We modified the WHO ordinal scale to these 4 points to avoid using hospitalisation as a criterion of severity because 1) many patients in nursing homes had severe infection but were not admitted to hospital, and 2) early in the epidemic, all cases were hospitalised irrespective of the severity of their infection. Our model included the presence of the D614G mutation and the biological sex of the patient as categorical predictors, as well as age and the time since the first case in the dataset as non-linear predictors. We include the time in days since the first case in the dataset to control for changes in treatment practice across the course of the epidemic. We mean-centered age and time in days and modeled their nonlinearities using penalised regression splines with a maximum of 30 knots. If a case was associated with a cluster of cases, for instance in a hospital ward or nursing home, this was included as a random effect with each cluster getting its own level. We gave any cases not associated with clusters their own unique level. Finally, to account for correlations driven by genome similarity that are not due to the D614G mutation, we generated a variance-covariance matrix (scaled to a correlation matrix) from the phylogeny described above (after dropping all tips corresponding to genomes not in the dataset) using the *ape* package v. 5.3 (Paradis and Schliep, 2019) and included that as a random effect in the model. We modeled the ordinal nature of the data using a cumulative model that assumes multiple thresholds corresponding to each severity level on the logit scale.

The model was fit in a Bayesian framework using Hamiltonian Monte Carlo in the R package *brms* v. 2.13.5 (Bürkner, 2018), a front-end for *rstan* v. 2.21.2 (Stan Development Team, 2020). The model had no divergent transitions, Gelman-Rubin values less than 1.01 and both bulk and effective sample sizes of greater than 950 for all parameters. Shortest probability intervals for reporting were generated by the R package *SPIn* v. 1.1 (Liu et al., 2015). We used weakly informative priors to constrain the model to sensible values on the link scale, but not rule out any reasonable values. All thresholds for the dividing lines between severity levels were given t-distribution (mean = 0, scale = 2.5, df = 3) priors and all fixed effects were given Gaussian (mean = 0, standard deviation = 2.5) priors. The standard deviations for the random effects and penalised splines were given Exponential (lambda = 0.4) priors, corresponding to a prior mean of the standard deviation of 2.5, the same as the fixed effects.
