# Supplementary material for: Evaluating the Effects of SARS-CoV-2 Spike Mutation D614G on Transmissibility and Pathogenicity
Source: Cell. 2021 Jan 7;184(1):64–75.e11. doi: 10.1016/j.cell.2020.11.020 (PMC7674007; doi:10.1016/j.cell.2020.11.020)
Supplement: Document S1. Tables S1–S3 [file mmc1.pdf]

Cell, Volume *184*

Supplemental Information

Evaluating the Effects of SARS-CoV-2 Spike Mutation D614G on Transmissibility and Pathogenicity

Erik Volz, Verity Hill, John T. McCrone, Anna Price, David Jorgensen, Áine O'Toole, Joel Southgate, Robert Johnson, Ben Jackson, Fabricia F. Nascimento, Sara M. Rey, Samuel M. Nicholls, Rachel M. Colquhoun, Ana da Silva Filipe, James Shepherd, David J. Pascall, Rajiv Shah, Natasha Jesudason, Kathy Li, Ruth Jarrett, Nicole Pacchiarini, Matthew Bull, Lily Geidelberg, Igor Siveroni, COG-UK Consortium, Ian Goodfellow, Nicholas J. Loman, Oliver G. Pybus, David L. Robertson, Emma C. Thomson, Andrew Rambaut, and Thomas R. Connor

Table S1 | Posterior values for regression coefficients and standard deviations for the severity analysis of Dataset 2, Related to Figure 3.

Effect	Posterior mean	Lower bound of 95% shortest probability interval	Upper bound of 95% shortest probability interval
Presence of 614G	0.03	-0.80	0.84
Female biological sex	-0.72	-0.95	-0.50
Threshold 1	0.07	-0.36	0.49
Threshold 2	0.92	0.50	1.34
Threshold 3	1.28	0.85	1.71
Cluster random effect standard deviation	0.26	0.00	0.58
Phylogenetic random effect standard deviation	0.62	0.19	1.09
Age penalised spline standard deviation	1.34	0.15	2.94
Days since first case in the dataset penalised spline standard deviation	5.98	2.39	10.27

Table S2 | Prior distributions used in the BEAST analysis (see Main text), Related to STAR Methods.

Parameter	Prior distribution	Shape parameters
Kappa (HKY)	Lognormal	Mu = 1.0 Sigma=1.25
Population size (for each lineage)	Lognormal	Mean = 1.0 Stdev = 1.5
Hyperprior for mean growth and decline rate	Normal	Mean = 0 Stdev = 100
Hyperprior for precision of growth and decline rate	Gamma	Shape = 1 Scale = 0.001
Transition time	Normal	Mean = 2020.2254 in node height of the individual cluster tree Stdev = 0.034

Table S3 | Early D614G viruses in lineage B, Related to Figure 1.

Name	GISAID ID	Date	Laboratories	Authors
Sichuan/SC-PHCC1-022	EPI_ISL_451345	2020-01-24	West China Hospital of Sichuan University / State Key Laboratory of Biotherapy of Sichuan University	Baowen Du, Minjin Wang, Chao Tang, Chuan Chen, Yongzhao Zhou, Mingxia Yu, Hancheng Wei, Weimin Li, Jing-wen Lin, Jia Geng, Binwu Ying, Lu Chen
Shanghai/SH0014	EPI_ISL_416327	2020-01-28	Shanghai Public Health Clinical Center, Shanghai Medical College, Fudan University / National Research Center for Translational Medicine (Shanghai), Ruijin Hospital affiliated to Shanghai Jiao Tong University School of Medicine & Shanghai Public Health Clinical Center	Shengyue Wang, Xiaonan Zhang, Gang Lu, Yun Tan, Yun Ling, Hongzhou Lu, Saijuan Chen
Shanghai/SH0086	EPI_ISL_416386	2020-01-31	Shanghai Public Health Clinical Center, Shanghai Medical College, Fudan University / National Research Center for Translational Medicine (Shanghai), Ruijin Hospital affiliated to Shanghai Jiao Tong University School of Medicine & Shanghai Public Health Clinical Center	Shengyue Wang, Xiaonan Zhang, Gang Lu, Yun Tan, Yun Ling, Hongzhou Lu, Saijuan Chen
Shanghai/SH0025	EPI_ISL_416334	2020-02-06	Shanghai Public Health Clinical Center, Shanghai Medical College, Fudan University / National Research Center for Translational Medicine (Shanghai), Ruijin Hospital affiliated to Shanghai Jiao Tong University School of Medicine & Shanghai Public Health Clinical Center	Shengyue Wang, Xiaonan Zhang, Gang Lu, Yun Tan, Yun Ling, Hongzhou Lu, Saijuan Chen
Guangzhou/GZMU0019	EPI_ISL_429080	2020-02-05	The First Affiliated Hospital of Guangzhou Medical University	Yanqun Wang, Daxi Wang, Lu Zhang, Wanying Sun, Zhaoyong Zhang et al.
Guangzhou/GZMU0037	EPI_ISL_429089	2020-02-08	The First Affiliated Hospital of Guangzhou Medical University	Yanqun Wang, Daxi Wang, Lu Zhang, Wanying Sun, Zhaoyong Zhang et al.
Guangzhou/GZMU0020	EPI_ISL_429081	2020-02-05	The First Affiliated Hospital of Guangzhou Medical University	Yanqun Wang, Daxi Wang, Lu Zhang, Wanying Sun, Zhaoyong Zhang et al.
Germany/BavPat1	EPI_ISL_406862	2020-01-28	Charité Universitätsmedizin Berlin, Institute of Virology; Institut für Mikrobiologie der Bundeswehr, Munich	Victor M Corman, Julia Schneider, Talitha Veith, Barbara Mühlemann, Markus Antwerpen, Christian Drosten, Roman Wölfel
